# The complete mitochondrial genome of *Octopus vulgaris*

**DOI:** 10.1007/s11033-023-08984-3

**Published:** 2023-12-18

**Authors:** Gareth N. Fee, Alvaro Roura, Arsalan Emami-Khoyi, Peter R. Teske

**Affiliations:** 1 Department of Biological Sciences, University of Cape Town, Cape Town, South Africa; 2 Departamento de Ecología y Biodiversidad Marina, Instituto de Investigaciones Marinas, Vigo, Spain; 3 Department of Zoology, University of Johannesburg, Auckland Park, South Africa

**Keywords:** *Octopus vulgaris*, Common octopus, Mitogenome, Northeastern Atlantic, Phylogeny, Cephalopoda

## Abstract

**Background:**

The *Octopus vulgaris* species complex consists of numerous morphologically similar but genetically distinct species. The current publicly available mitogenome of this species has been generated from a specimen collected from Tsukiji Fish Market, Tokyo, Japan. Octopus from the northwestern Pacific Ocean are now considered to be a separate species, *Octopus sinensis*. For this reason, we hypothesised that the current record of *O. vulgaris* was sequenced from a specimen of *O. sinensis*. Here, we sequenced the first complete mitogenome of a specimen of *Octopus vulgaris *sensu stricto that was collected from the species’ confirmed distribution areas in northeastern Atlantic.

**Methods and results:**

The complete mitogenome was assembled de novo and annotated using 250 bp paired-end sequences. A single circular contig 15,655 bp in length with a mean read coverage of 1089 reads was reconstructed. The annotation pipeline identified 13 protein-coding genes (PCGs), 22 transfer RNAs (tRNA) and two ribosomal RNAs. A maximum likelihood phylogenetic tree recovered the assembled mitogenome as the sister taxon of a monophyletic group comprising *O. sinensis* and the previously published mitogenome of “*O. vulgaris*” from Japan. This confirms that the latter was a Japanese specimen of *O. sinensis*.

**Conclusion:**

The mitogenome sequenced here is the first to be published for *Octopus vulgaris *sensu stricto. It represents an important first step in genetics-informed research on the evolution, conservation, and management of this commercially important species.

## Introduction

The family Octopodidae d'Orbigny, 1840 consists of over 200 known species, many of which lack detailed taxonomic descriptions [1]. The taxonomy and phylogenetic relationships within this family have received much attention in recent years, with many cryptic species being identified across the globe [2–5]. A taxonomic group which has recently gained particular attention is the *Octopus vulgaris* species complex. Once thought to be a cosmopolitan species in temperate and tropical waters [6], *O. vulgaris *sensu stricto (*s.s.*) is now considered to be restricted to the Mediterranean and the northeastern Atlantic, while locations beyond this region are inhabited by morphologically similar but genetically distinct species [1, 7], some of which may still need to be formally described. As a case in point, the scientific name *Octopus sinensis* was recently reinstated for the East Asian common octopus previously referred to as *O. vulgaris*, based on a combination of molecular and morphological differences [8], while the Western Atlantic lineage was found to contain two more species, *Octopus americanus* [4] and *Octopus insularis* [3].

*Octopus vulgaris* is the most important commercially exploited octopus species worldwide, and off northwest Africa, it is the target of the world’s largest single-species octopus fishery. In 2010, total octopus catches for the northwest coast of Africa and Europe were 57,982 and 42,945 tonnes, respectively, consisting largely of *O. vulgaris* [9]*.* Currently, the poor state of octopus taxonomy is the single largest impediment to accurate catch statistics and comprehensive management plans for octopus species worldwide [9].

Mitochondrial DNA is a useful genetic marker for phylogenetic studies due to its maternal inheritance, conserved gene arrangement and comparatively high mutation rate [10]. Mitogenomes have been used to investigate deep phylogenetic relationships across different taxa [11], as well as delineate closely related species [12].

Within the *O. vulgaris* species complex, two complete mitochondrial genomes are currently available on the public databases: “*O. vulgaris”* (NC_006353.1 [13]) and *O. sinensis* (NC_052881.1 [14]). However, the original specimen used to create the first publicly available *O. vulgaris* mitogenome (NC_006353.1) was collected at the Tsukiji Fish Market, Tokyo, Japan in the early 2000s [13], before the scientific name *O. sinensis* was reinstated [8]. Since *O. sinensis* is distributed in the NW Pacific Ocean (from northern Japan to Taiwan), we hypothesised that this record in fact corresponds to *O. sinensis*. This suggests that despite its commercial and ecological importance, not a single complete mitogenome has yet been reconstructed for *O. vulgaris s.s.* The objective of the current study was to assemble, annotate, and describe the mitochondrial genome of *O. vulgaris s.s.* collected from its confirmed distribution range within northeastern Atlantic waters, and to examine its phylogenetic placement among closely related species. This study is important to improve our understanding of the taxonomic and phylogenetic relationships within the *O. vulgaris* species complex.

## Material and methods

### Sample collection, genomic library preparation and sequencing

The tissue sample of *O. vulgaris s.s.* was obtained from a specimen that was commercially caught near the Cies Islands in the Illas Atlanticas de Galicia National Park, Spain, in August 2022. DNA of high molecular weight was extracted from a small piece of muscle tissue using the QIAGEN DNeasy Blood & Tissue kit (Hilden, Germany). A genomic library was constructed from the extracted DNA using the NOVO kit (Novogene, Beijing, PRC). For this purpose, the DNA was first sheared into smaller fragments, and fragments of size ~ 350 bp were selected for the adaptor ligation step. The quality of the genomic library was checked using a combination of Qubit (Thermo Fisher Scientific, Waltham, USA), qPCR, and the DNA NGS 3 K assay (PerkinElmer, Waltham, USA). The quality-checked genomic library was sequenced on a NovaSeq 6000 SP platform (Illumina, San Diego, USA) using the paired-end 250 protocol.

### Mitogenome assembly and annotation

The mitochondrial genome was assembled de novo using the GetOrganelle v.1.7 assembly pipeline [15]. The assembly parameters were set to their defaults, except for the kmer values, which were set for a combination of the following: 21, 45, 65, 85 and 105. The assembled mitogenome was then submitted to the MITOS Web Server [16] for annotation. The predicted gene boundaries were manually adjusted in MEGA11 [17] using the mitogenomes of the previously published “*O. vulgaris*” from Japan (NC_006353.1) and *O. sinensis* from China (NC_052881.1) as template references. The nucleotide composition of the complete mitogenome was calculated manually using the formulas AT-skew = (A − T)/(A + T), and GC-skew = (G − C)/(G + C) [18]. The annotated mitogenome was visualised in Chloroplot [19].

### Maximum likelihood phylogenomic analysis

To reconstruct the phylogenetic relationships between the mitogenome of European *O. vulgaris s.s.* and those from other octopus taxa, the assembled mitochondrial genome was blast-searched against the NCBI nucleotide database. The complete mitogenomes of eight closely related species of octopus, as well as an outgroup species, *Tremoctopus violaceus*, were retrieved for phylogenetic analysis. The Ezsplit tool [20] was used to extract the sequences of all 13 protein coding genes (PCG) from the NCBI database, each of which was aligned separately using the codon alignment option in ClustalW with default setting [21].

A maximum likelihood phylogenetic tree was then constructed using IQ-TREE [22]. The most suitable evolutionary model was identified with ModelFinder [23]. Branch support was assessed using the ultrafast bootstrap analysis [24] based on 1000 bootstrap alignments. All other parameters were set to default. The resulting tree was visualised in Figtree v.1.4.3 [http://tree.bio.ed.ac.uk/software/figtree/].

## Results and discussion

**Fig. 1:**
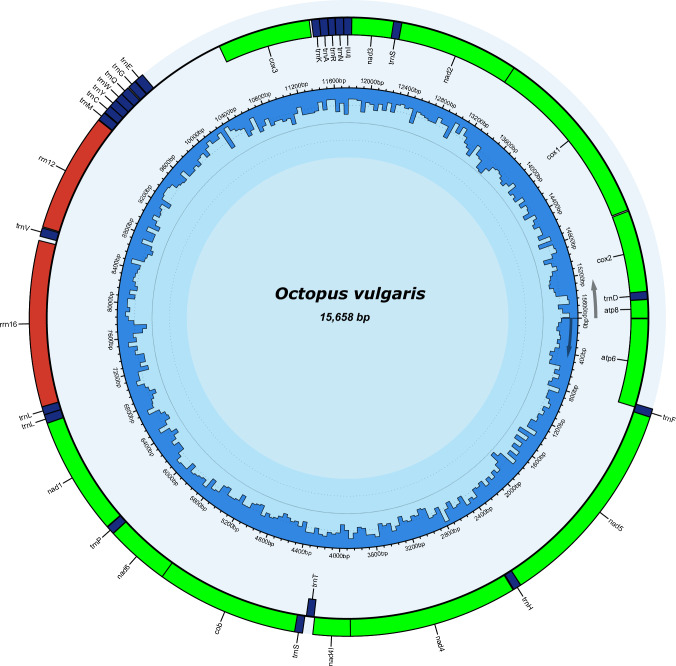
Graphic representation of the European *Octopus vulgaris s.s.* mitogenome, indicating the location of tRNAs (blue), rRNAs (red), and protein coding genes (green). Blue bars represent GC content. (Color fogure online)

In total, the sequencing runs produced 10.4 million paired-end sequences with an average Phred quality score of 36. De novo assembly of the sequences produced a single circular contig 15,655 bp in length, with an average coverage of 1089, and a GC content of 25% (Fig. 1). The mitochondrial genome has a positive AT skew (0.096) and a positive CG skew (0.392) with base frequencies of A = 41.1%, C = 17.4%, G = 7.6% and T = 33.9%. The MITOS annotation pipeline identified 13 protein-coding genes (PCGs), 22 transfer RNAs (tRNA) and two ribosomal RNAs (rRNA) consistent with those reported from other octopus species. The plus strand contained seven PCGs (atp6, atp8, cox1, cox2, cox3, nad2, nad3) and eight tRNAs while the minus strand contained 6 PCGs (cytb, nad1, nad4, nad4l, nad5, nad6), 14 tRNAs and 2 rRNAs. The PCGs start with ATG, except for nad4, which has ATA as its start codon. The annotation pipeline also identified a single long intergenic sequence of approximately 645 bp between trnE(gaa) and cox3, which corresponds to the control region. Several (n = 21) shorter intergenic sequences ranging in length from 1 to 52 bp were also found.

The NCBI blast results showed European *Octopus vulgaris s.s.* to be most closely related to the only two other representatives of the *O. vulgaris* species complex with published mitogenomes. These are *O. sinensis* from China (NC_052881.1) and “*O. vulgaris*” from Japan (NC_006353.1), with 96.33% and 96.25% identity, respectively. Among other publicly available complete mitochondrial genomes, the next closest matches were *O. bimaculoides* (NC_029723.1) and *O. mimus* (NC_044093.1), each with roughly 85% identity.

**Fig. 2:**
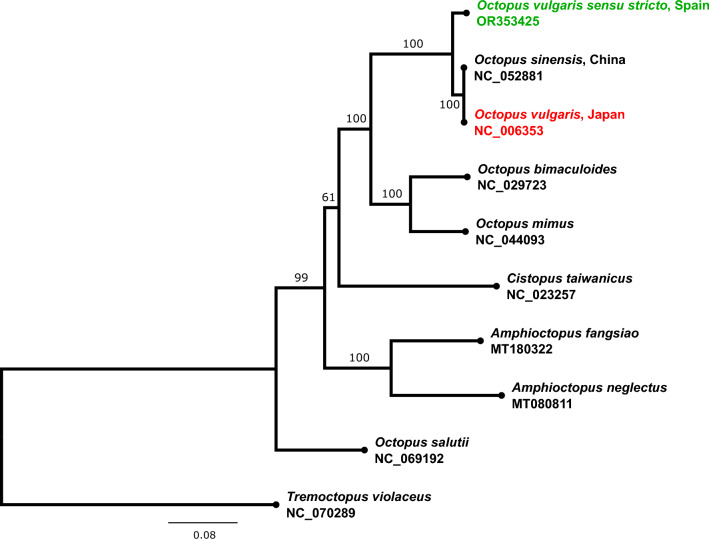
A maximum likelihood phylogenetic tree reconstructed using protein coding genes under the TVM + F + I + G4 model, with a proportion of invariable sites of 0.51 and a gamma distribution shape parameter (α) of 1.54. The new mitogenome produced from the European specimen is shown in green, and that of the misidentified specimen that represents a second specimen of *Octopus sinensis* is shown in red. Bootstrap support values are shown to the left of the nodes. (Color fogure online)

The maximum likelihood phylogenetic tree confirmed, with 100% bootstrap support, that the assembled mitogenome is distinct from that of both *O. sinensis* from China and from the Japanese specimen [6] that has been deposited in the NCBI database as *O. vulgaris* (NC_006353.1), but it forms a monophyletic group with these specimens (Fig. 2). A blast search of the Japanese specimen showed 99.85% identity to the mitogenome of *O. sinensis* from China [14], but only 96.25% to that of the European *O. vulgaris s.s.* generated in this study. This confirms our hypothesis that the mitochondrial sequence from the Japanese specimen represents an additional mitogenome of *O. sinensis*. The present study thus reports the first ever mitogenome of *O. vulgaris s.s.*

## Conclusion

Recent advances in DNA sequencing technology and high throughput computation have made it possible to assemble large numbers of mitogenomes. However, this information can only be useful in resolving taxonomic uncertainties when the specimens were correctly identified [25]; to this end, existing records need to be revisited to reflect the latest developments in how species are classified.

The phylogenetic relationships within the family Octopodidae are an ongoing topic of investigation. Mitogenomic evidence in this study shows that the current record of an “*O. vulgaris”* mitogenome in the NCBI database represents mitochondrial sequences from *O. sinensis*. Overall, our findings contribute towards improving our understanding of octopus phylogeny and taxonomy, which can ultimately inform fisheries management and improve the accuracy of catch statistics. The generation of additional mitogenomes from the broader geographical region inhabited by species within the *O. vulgaris* species complex will further enhance this field of knowledge.

## Data Availability

The annotated mitogenome of *Octopus vulgaris s.s.* has been submitted to the NCBI nucleotide database under accession number OR353425.
